# Supplementary material for: Whisker-based pre-neuronal and peripheral encoding of surface stickiness
Source: bioRxiv. 2026 May 14:2026.05.11.724292. Preprint. [Version 1] doi: 10.64898/2026.05.11.724292 (PMC13192646; doi:10.64898/2026.05.11.724292)
Supplement: 1 [file NIHPP2026.05.11.724292V1-supplement-1.pdf]

bioRxiv preprint doi: https://doi.org/10.64898/2026.05.11.724292; this version posted May 14, 2026. The copyright holder for this preprint (which was not certified by peer review) is the author/funder, who has granted bioRxiv a license to display the preprint in perpetuity. It is made available under a CC-BY 4.0 International license.

Supplemental Information

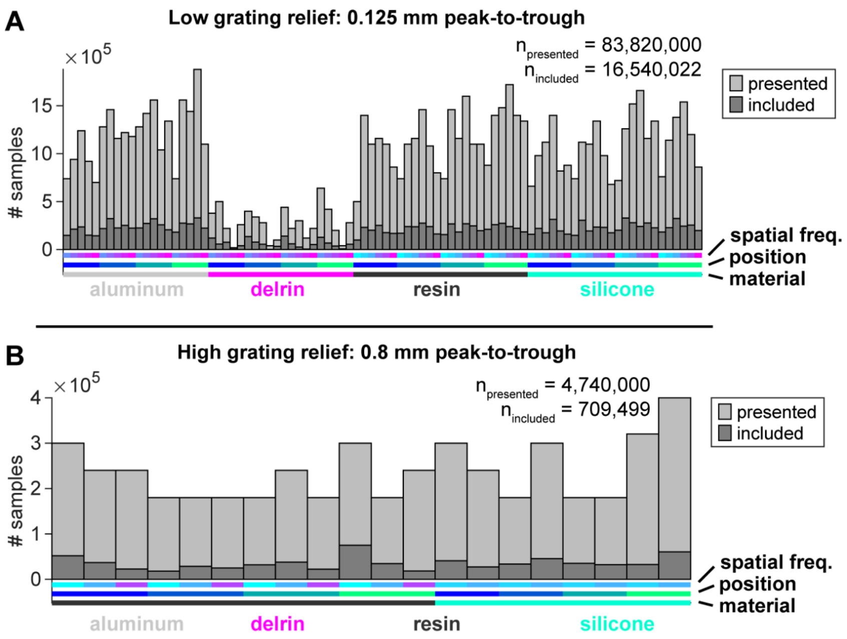

Figure S1 – Samples were evenly distributed across experimental conditions, with the exception of Delrin as least-encountered material.
A, B) Samples (frame-wise observations of single whisker instances) observed in each texture state, pooled across all sessions in which the original low-relief gratings (A) and the supplementary high-relief gratings (B) were used. “Included” samples are those passing the three inclusion criteria of surface contact, active whisking, and absence of outlier whisker strain or kinematics.

bioRxiv preprint doi: https://doi.org/10.64898/2026.05.11.724292; this version posted May 14, 2026. The copyright holder for this preprint (which was not certified by peer review) is the author/funder, who has granted bioRxiv a license to display the preprint in perpetuity. It is made available under a CC-BY 4.0 International license.

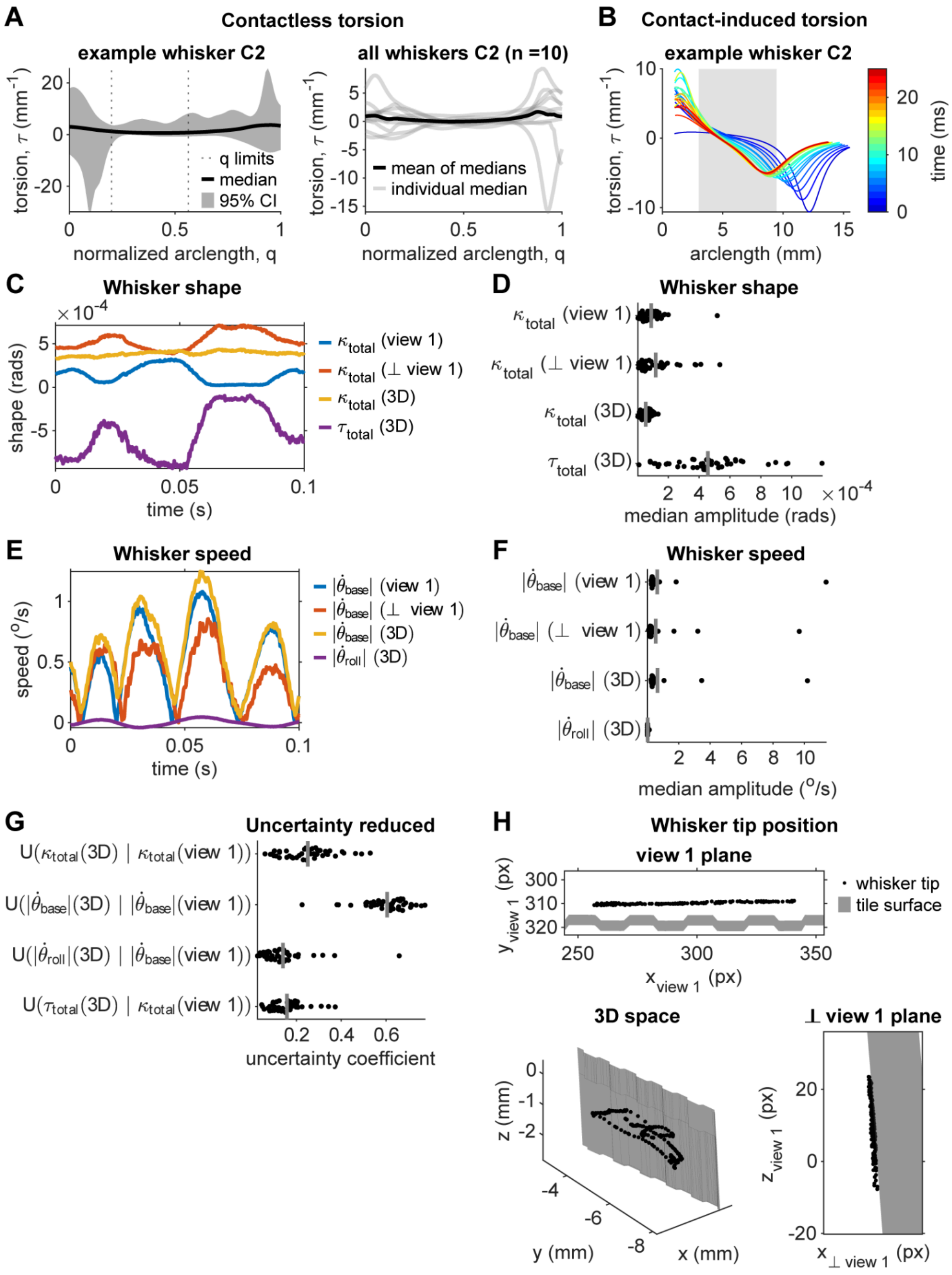

Figure S2 – Information about whisker shape and motion during whisking against surfaces is lost when tracking is limited to a top-down view.
A) Torsion in the shaft of a single example C2 whisker (left) and the full set of C2 whiskers (right), without contact or whisking. **B**) Torsion in the shaft of a single example C2 whisker over a 25 ms period of contact and whisking (with 5x temporal downsampling for visualization). **C**) Example 2D and 3D whisker shape signals measured during a 0.1 s period of contact and whisking. **D**) Median amplitudes of the whisker shape signals for all whisker instances. **E**) Example 2D and 3D whisker motion signals measured during the same period of contact and whisking as in C. **F**) Median amplitudes of the whisker motion signals for all whisker instances. **G**) Uncertainty coefficients between 3D whisker shape and motion signals and their 2D comparators. **H**) Position of the whisker tip over the same time period as in C and E, at 2x temporal downsampling.

bioRxiv preprint doi: https://doi.org/10.64898/2026.05.11.724292; this version posted May 14, 2026. The copyright holder for this preprint (which was not certified by peer review) is the author/funder, who has granted bioRxiv a license to display the preprint in perpetuity. It is made available under a CC-BY 4.0 International license.

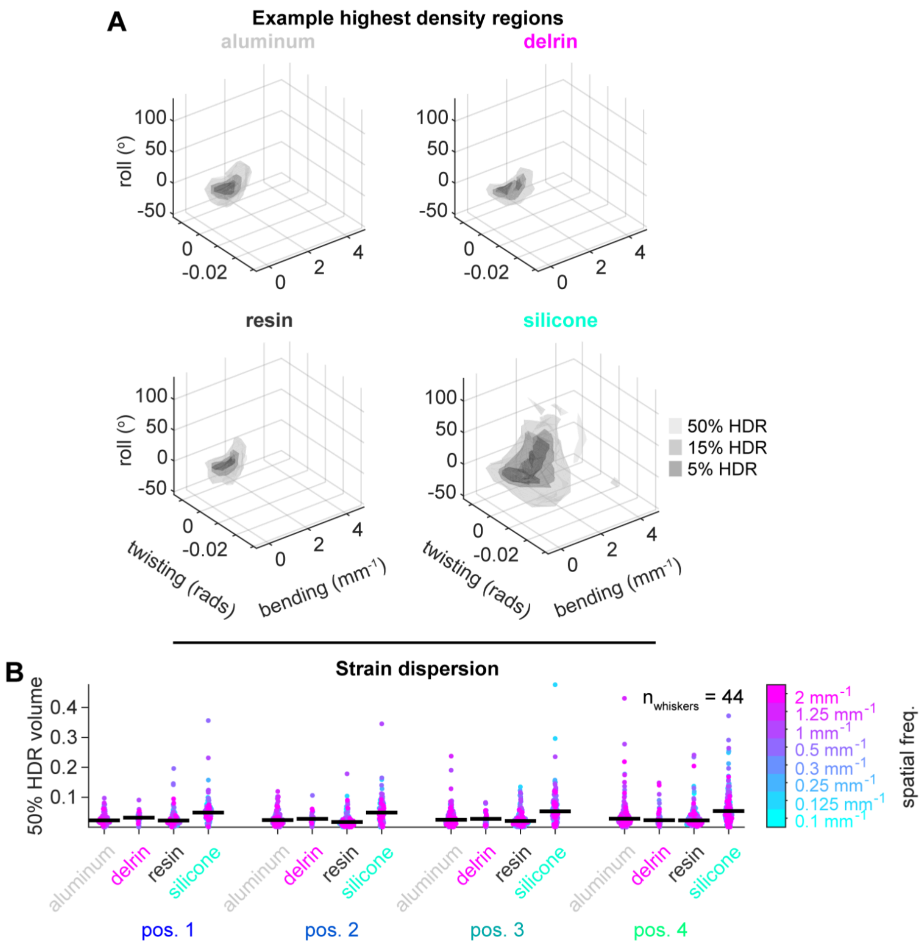

Figure S3 – Highest density regions of the whisker deformation distribution.
A) Gray volumes indicate the boundaries of the 50%, 15%, and 5% highest density regions of the example distributions shown in Figure 3B. **B)** Scatter plots of the 50% highest density region volumes of all whisker instances' deformation distributions; points are grouped by position, subgrouped by material, and color coded by frequency. Each point corresponds to a single whisker instance that was observed under the conditions indicated.

bioRxiv preprint doi: https://doi.org/10.64898/2026.05.11.724292; this version posted May 14, 2026. The copyright holder for this preprint (which was not certified by peer review) is the author/funder, who has granted bioRxiv a license to display the preprint in perpetuity. It is made available under a CC-BY 4.0 International license.

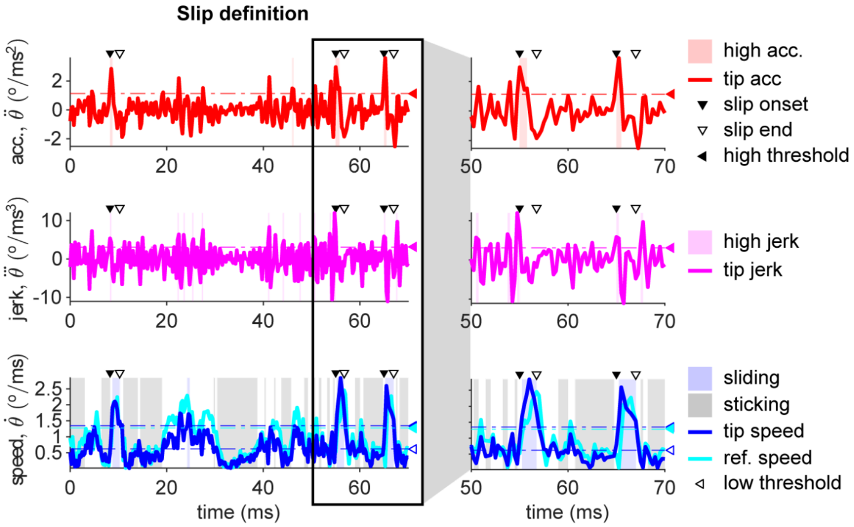

Figure S4 – Stick-slip event boundaries were extracted from the kinematics of the whisker tip and reference point.
Whisker tip acceleration (top), whisker tip jerk (middle), and whisker tip and reference point speed (bottom) traces over a random interval containing multiple slips. Filled and unfilled triangles above each panel indicate the start and end times, respectively, of the detected stick-slip events. Filled (all panels) and unfilled (bottom only) triangles to the right of each panel mark the high and low thresholds, respectively, for the plotted variables. Shading indicates frames classed as high acceleration, high jerk, sliding, or sticking, based on threshold crossings. Inset at right zooms in on two slips.

bioRxiv preprint doi: https://doi.org/10.64898/2026.05.11.724292; this version posted May 14, 2026. The copyright holder for this preprint (which was not certified by peer review) is the author/funder, who has granted bioRxiv a license to display the preprint in perpetuity. It is made available under a CC-BY 4.0 International license.

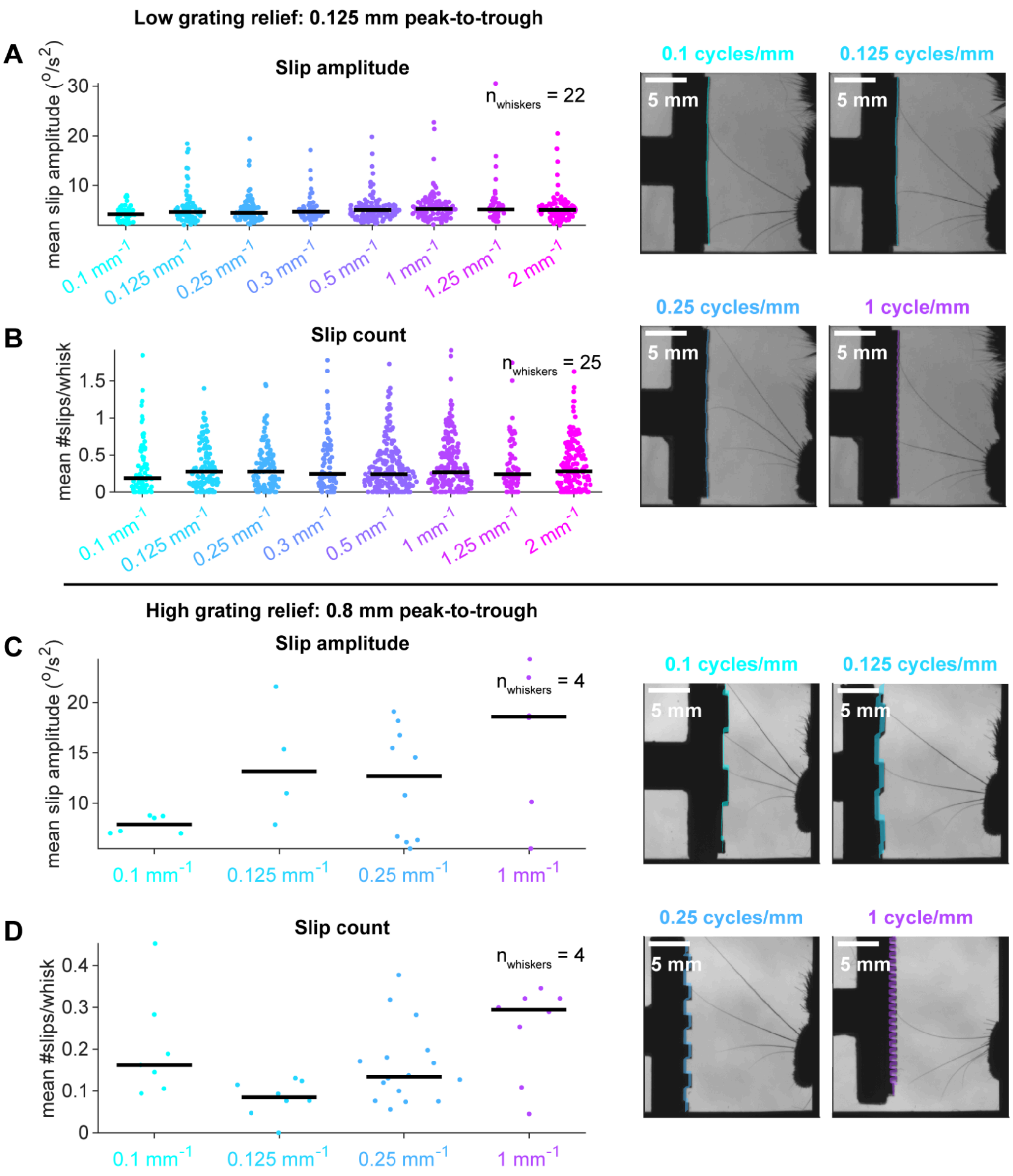

Figure S5 – Relationships of stick-slip amplitude and rate to spatial frequency were weak in both high and low grating relief conditions.

A, B) Scatter plots of mean slip amplitude (A) and rate (B), grouped by frequency, for the low-relief set of gratings. **C, D)** Scatter plots of mean slip amplitude (C) and rate (D), grouped by frequency, for the high-relief set of gratings. As in Figures 3 and 4, each point corresponds to a single whisker instance observed under a single experimental condition.

bioRxiv preprint doi: https://doi.org/10.64898/2026.05.11.724292; this version posted May 14, 2026. The copyright holder for this preprint (which was not certified by peer review) is the author/funder, who has granted bioRxiv a license to display the preprint in perpetuity. It is made available under a CC-BY 4.0 International license.

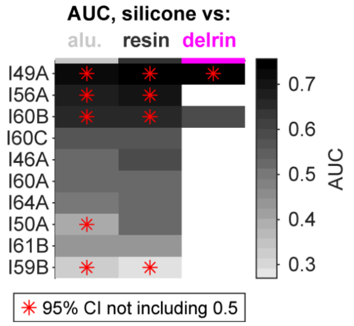

Figure S6 – Whisks against silicone were discriminable from other materials on the basis of single-unit response.
Heatmap displays, for each unit, the AUC for discrimination of silicone from each other material the unit encountered.

bioRxiv preprint doi: https://doi.org/10.64898/2026.05.11.724292; this version posted May 14, 2026. The copyright holder for this preprint (which was not certified by peer review) is the author/funder, who has granted bioRxiv a license to display the preprint in perpetuity. It is made available under a CC-BY 4.0 International license.

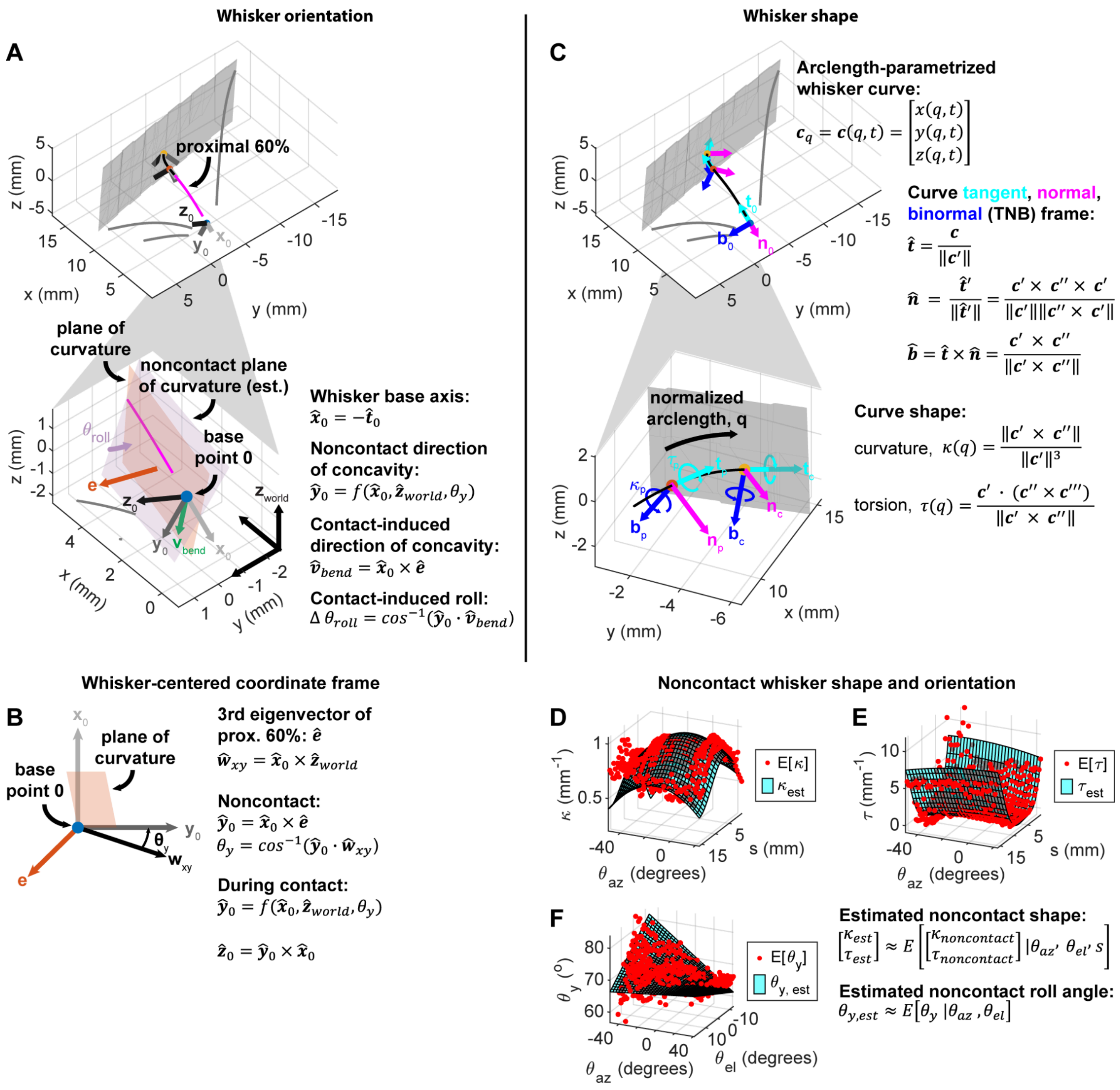

Figure S7 – Computation of whisker shape, strain, and orientation.
A) Contact-induced whisker roll was defined as the angle between the instantaneous plane of curvature of the proximal 60% of the whisker shaft (orange), and the estimated noncontact plane of curvature of the proximal 60% of the whisker shaft (purple). **B)** The whisker's estimated noncontact plane of curvature defined the directions of the y and z basis vectors of the whisker-centered coordinate frame. **C)** The whisker curve's tangent, normal, binormal (TNB) frame at each point along its length was computed from the curve and its first three derivatives. The curve's shape (curvature and torsion) was the angular velocity of the TNB frame with respect to normalized arclength. **D-E)** The curvature (D) and torsion (E) of the undeflected whisker, given arclength along the whisker and the position (azimuth and elevation angles) of the whisker base, were estimated as fifth-order polynomial surfaces (cyan meshes) fitted to the average values observed during low-acceleration whisking without contact (red dots). Elevation angle is limited to a single value for visualization. **F)** The angle between the undeflected proximal whisker segment's plane of curvature and the world x-y plane, given the whisker's

bioRxiv preprint doi: https://doi.org/10.64898/2026.05.11.724292; this version posted May 14, 2026. The copyright holder for this preprint (which was not certified by peer review) is the author/funder, who has granted bioRxiv a license to display the preprint in perpetuity. It is made available under a CC-BY 4.0 International license.

position, was estimated as a quadratic surface (cyan mesh) fitted to the average values observed during low-acceleration whisking without contact (red dots).
